# The meaning of marriage, the meaning of family, and the function of family for Indonesian married people

**DOI:** 10.1186/s40359-025-03670-4

**Published:** 2025-11-25

**Authors:** Langgersari Elsari Novianti, Fredrick Dermawan Purba, Stella Vania Puspitasari, Agfa Aghnia Nadirah, Ita Novita Br Purba, Johan C. Karremans, Hendriati Agustiani

**Affiliations:** 1 Faculty of Psychology, Universitas Padjadjaran, Sumedang, Indonesia; 2 Behavioural Science Institute, Radboud University, Nijmegen, Netherlands; 3 Center for Relationship, Family Life, and Parenting Studies, Faculty of Psychology, Universitas Padjadjaran, Sumedang, Indonesia; 4 Center for Psychological Innovation and Research, Universitas Padjadjaran, Sumedang, Indonesia

**Keywords:** Meaning of marriage, Meaning of family, Function of family, Indonesia

## Abstract

**Background:**

While marriage is the most common relationship arrangement across the globe, its meaning can vary between individuals, countries, and also within a country. Indonesia is a culturally diverse country, so the current study aims to explore whether this diversity is reflected in different views on marriage or whether a culturally diverse of Indonesians has similar views on marriage and family.

**Methods:**

We conducted a descriptive exploratory qualitative study using online structured interviews lasting 60–90 min. The data from the structured interviews were processed using thematic analysis. We obtained data from 20 married participants from diverse cultural backgrounds, aged 25–50 years, with various lengths of marriage, educational backgrounds, occupations, and places of residence.

**Results:**

It showed that the meaning of marriage is that the family becomes one and has the same vision or goal. Most participants mentioned the meaning of family as having blood or marriage ties and family as a source of strength that can provide positive energy for its members. Participants also mentioned love, care, protection, help, sharing and support for each family member as the function of the family.

**Conclusion:**

The findings highlighted several consistent themes with regard to the meaning of marriage as well as the meaning and function of family. As one of the first studies using a qualitative approach among a diverse sample in Indonesia, the current findings suggest that Indonesians from different cultures and backgrounds share similar views on the meaning of marriage, the meaning of family, and the function of family. As compared to previous studies in other cultures, there were some unique views and opinions that seemed to reflect the inherent collectivist culture participants are embedded in. We discuss the implications of our findings for the study of marriage and family in Indonesia as well as the broader implications of the current findings.

**Supplementary Information:**

The online version contains supplementary material available at 10.1186/s40359-025-03670-4.

## Background

Marriage is considered one of the developmental tasks of young adults [1], usually taking place between the ages of 24 and 34 [1]. Most people in this age group have completed secondary education and have their own income. In Indonesia, these two conditions are considered to be characteristics of readiness for marriage. Other developmental tasks generally considered in Indonesia include becoming a father or mother, carrying out the role of breadwinner for men, taking care of the house for women, and becoming independent from one’s parents. Notably, across various countries, there has been an increase in the average age of marriage over the past few decades [2–4].

The study of marital relationships continues to be of interest (e.g., Berger & Kellner [5], Cherlin [6], Katz [7]) and is regularly investigated in various countries. Perceptions of marriage and family life are one such area of study, as these perceptions appear to be related to marital outcomes [8]. Giving a positive meaning to marriage (for example, by contemplating the benefits of marriage or having a positive philosophy of marriage and a positive perception about marriage and family) can reduce the risk of divorce in married couples [8, 9]. Marriage can also be perceived in terms of moral and structural commitment, and one of the indications of this commitment is childbearing and joint mortgages [10]. Research has shown that individual perceptions about marriage are influenced through social norms, family experiences, and own experiences in romantic relationships, before and during marriage [11]. Moreover, an increasingly prominent factor that can shape perceptions about marriage is engagement with social media [12, 13]. A study in Indonesia found that social media use can undermine traditional views on marriage [14].

In Indonesia, in addition to a bond between man and woman, marriage is also an institution for raising children who will be legally recognized, whereas the presence of children outside marriage is contrary to social values and norms. The Indonesian government defines marriage as “the relationship between men and women, with a division of roles as husband and wife, aimed at forming a long-lasting happy family on the basis of God Almighty” and legalizes marriage at the age of the 19 [15]. In Indonesia, only marriage between a man and a woman is legally recognized. Half of Indonesians aged 25–49 have been married [16, 17], almost 30% people in this age group have completed senior high school and have their own income [18]. Previous research in Indonesia showed that these two conditions are considered to be characteristics of readiness for marriage [19]. Other criteria for ‘marriage readiness’ include being capable of caring for children as a father or mother, being biologically capable of fathering or bearing children, being able to take on the role of breadwinner for men, taking care of the home for women, becoming independent from one’s parents [20, 21], and having sufficient knowledge about maintaining a family [21, 22]. Another study [23] showed that the readiness of marriage include age readiness, and having sufficient physical, financial, mental, emotional, social, moral, interpersonal, intellectual, and life skills.

Indonesia is the largest archipelago country in the world, with more than 300 ethnic groups [24] maintaining different traditions and lifestyles spread across 17,000 islands. With 718 regional languages [25], six formally recognized religions and various local beliefs [26], and 38 provinces, it is a very multiculturally diverse country. Although intercultural marriage is possible in Indonesia, it is generally considered that different perspectives on marriage and family life from diverse cultural backgrounds might complicate marital adjustment [27–31]. We are interested to know whether people from different cultural backgrounds in Indonesia have similar or different views on marriage and family.

Researchers have conducted several studies on marriage in relation to specific ethnic cultures in Indonesia (see Dhofier [32] and Smith-Hefner [33] for Javanese; Sari et al. [34] for Sundanese; and Sitompul et al. [35] for Batak). Previous investigations on the meaning of marriage among Indonesian people have examined and revealed several aspects of marriage. For example, marriage is seen as a new stage of life that one must prepare for; it requires commitment and responsibility, as well as partnership, and must be taken seriously [36]. Moreover, marriage is seen as a relationship to be managed for life, one in which the husband and wife must have mutual feelings; marital ties must be made legal by law and religion and also recognized by society [37]. Also, in marriage, the husband and wife will work together, complement each other, understand each other, and protect each other [38]. Marriage is viewed as an institution for continuing offspring as determined by God, a process that involves positive and challenging interactions within the family, and an expression of affection [39]. Making the decision to get married means that one has grown up and that at some point in time marriage is a life choice [40]. Notably, singlehood in Indonesia is increasing and many factors contribute to the increasing favorability of a single lifestyle; however, most Indonesian singles are still desiring marriage [41].

Previous studies on the meaning of marriage in Indonesia were conducted on groups of participants that were situation-specific and homogeneous. Examples include women married to blind men [39], millennials (born 1982–2000) choosing not to marry or delaying marriage [36], individuals marrying as teenagers under the age of 18 [38, 40], wives filing for divorce from their husbands [42], and women who remarried [37]. Similar studies around the world, such as in Switzerland [43], Turkey [44], and the US [45, 46], have also examined the meaning of marriage in a specific sample group, for example, men who are married or cohabiting in the transition to fatherhood, college seniors, black Americans, and married black men, respectively.

Studies on the meaning of marriage in Indonesia rarely include the perspectives of married people and are rarely conducted across different ethnic, religious, age, and educational backgrounds at the same time. In this study, we are interested in discovering the perceptions of marriage among Indonesian people from different backgrounds. This study aims to explore the possibility of the existence of similarities in perceptions of marriage, meaning of family, and family functions across different cultures and backgrounds in Indonesian society. This is important to study for both theoretical and applied reasons. It is a theoretically interesting question whether and how national diversity in cultural background may shape views on marriage. For applied reasons, it is interesting to know whether Indonesians generally have similar or very different views on marriage depending on their specific cultural background, which has applied value in the area of marriage counseling or relationship therapy (e.g. knowledge about the specific cultural background and views on marriage associated with it may help counselors).

In addition to exploring the meaning of marriage, the current study we also explores the views people have on the family. When people talk about marriage, they often discuss their current family that was formed after they got married, their feelings, and what family members do in marital-family relationships. Through marriage, couples become united as a family, forming a new system [1, 47] separate from their family of origin. Cox and Demmitt [48] define family as whatever system a society uses to support and control human sexual interaction, reproduction, and child rearing. Two previous studies in Indonesia, using specific samples (millennial adolescents [49]; adolescent children of divorced parents [50]) mentioned the importance of the family as a safe and comfortable place to grow, a place to love each other, and a place for all family members to experience togetherness.

In addition to research on the meaning of marriage, previous studies explored perceptions of the meaning of family. Duval and Miller [47] stated that the family has several functions; for example, the family is a source of affection (generating affection), provides personal security and acceptance, offers satisfaction and a sense of purpose, and teaches discipline/control. Patterson [51] described a similar distinction of family functions, namely, membership and family formation, economic support, nurturance, education, socialization, and protection of vulnerable members. Studies on family functions in Indonesia mostly examine eight family functions, according to the Indonesian Ministry of Social Affairs, including the functions of love, protection, reproduction, socialization, education, economic, socio-cultural, religious, and environmental development (e.g., Aswarna [52] and Herawati et al. [53]). Other studies applied quantitative methods to investigate the correlation between family functions and quality of life (e.g., Anggraini [54], Oktowaty et al. [55], Putri and Permana [56], and Sutikno [57]).

The present study aimed to explore perceptions about the meaning of marriage, the meaning of family, and the functions of family in a sample of Indonesian married couples. We explored whether individuals in a sample of diverse socio-demographic backgrounds (e.g., age, gender, religion, ethnicity, and educational background) would have similar or diverse perceptions on meanings about marriage and family and the functions of family. Exploration of these issues among Indonesian participants with diverse cultural backgrounds could provide new insights into whether the meaning of marriage and family is culturally shared.

## Method

This is a descriptive exploratory qualitative research [58, 59], which is considered an appropriate methodology for the purpose of summarizing, understanding, describing, and exploring the phenomenon [58, 59]. The study was part of a larger study on Family Resilience approved by the Ethics Committee of Universitas Padjadjaran under number 1182/UN6.KEP/EC/2020. Informed consent was obtained verbally from all subjects before the interview session.

### Participants and recruitment

In this study, we investigated the meaning of marriage as well as the meaning and function of family according to married participants. A purposive sampling approach was applied [60], with the following inclusion criteria: (1) heterosexually married, according to Indonesian marriage law (2) living with their partner in the same house, (3) minimum aged 19 years old (according to the government regulation on minimum age for marriage), and (4) able to use Google Meet video conferencing during the interview session. We used online interviews, because during the study there was an outbreak of Covid-19 in Indonesia and the government ordered social restrictions, closed all schools, offices, and many public places.

To recruit prospective participants, we broadcasted information about the research to various communities through Instant Messenger and social media applications for one week (January 21—28, 2021), and attached a Google Form link registration. A total of 146 informed prospective participants entered their personal data into the Google Form. We reviewed the demographic background and selected 23 participants representing a diverse range of ages, length of marriage, educational backgrounds, occupations, place of residence, religions, and ethnicities. We observed and took into account the diverse backgrounds of the participants who applied to participate in the study and decided that the participants we interviewed should represent Islam, Catholicism, Christianity, Buddhism and Hinduism; represent high school, undergraduate and postgraduate levels; represent diversity of ethnicity; represent newly married, married for 5–10 years, 10–15 years and after 15 years; and represent diversity of place of residence (living in Sumatra Island, Java Island, Bali).

Three of the 23 people were randomly selected to be participants in the pilot interview and another 20 were interviewed for the final data collection. From the 20 final participants, there were five selected participants who could not continue their participation in this study for the following reasons: (1) schedule mismatch, (2) commuter marriage, (3) medical treatment with a psychiatrist. This was done after being re-checked by the interviewer. These participants were excluded because they did not meet the eligibility criteria, or because the researchers feel that their well-being was at risk. The five omitted participants were replaced by those listed in the potential participants with similar characteristics of age, length of marriage, religion, and educational background.

The final sample consisted of 20 participants aged 25 to 50 years (mean age = 35.15); eight participants had been married for less than two years, three for 3–5 years, four for 5–10 years, and five for more than 10 years. Their occupations were teacher/lecturer, psychologist, entrepreneur, nurse, doctor, pastor, employee in private company, and civil servant. Their educational backgrounds were senior high school (*n* = 1), associate degree (*n* = 1), undergraduate degree (*n* = 6), professional program (*n* = 2), master’s degree (*n* = 8), and doctoral degree (*n* = 2). Fifteen participants had children; the rest had no children. Participants came from various provinces in Indonesia, from Sumatera, Java, and Bali. They were Muslim, Protestant, Catholic, Hindu, and Buddhist. Regarding ethnicity, six participants were Javanese, four were Sundanese, four were Batak, three were from Bali, one was ethnic Chinese, one was Minahasa, and one was Sumbawa.

### Interview procedure

Four members of the research team (authors 1, 3, 4, 5), all of whom were psychologists, conducted the interviews in February 2021; each interviewed five participants. Before the interview, the interviewer contacted the participants via WhatsApp chat to get acquainted and confirm each participant’s willingness to participate. After the participant confirmed his/her willingness to participate, the interviewer provided a Google Form link containing a list of items that the participant had to prepare for the interview session (e.g., a closed room where the participant was alone so that he/she could express his/her experiences and opinions freely; good internet signal) and the interview schedule that the participant preferred. The interviewer then contacted the participant again to confirm the schedule and provided a Google Meet link for the online meeting. The overall interview duration was 60 min, conducted in Bahasa Indonesia. The questions developed by the researchers to be discussed with the participants (see the interview guide on supplement) consisted of three questions:

1. What is the meaning of marriage for you?
2. What is the meaning of family for you? Who do you think we can call family?
3. What are the functions of the family? What must the family do for its members?

Follow-up questions were asked to elicit the experience of the participant in greater depth. The interview process was recorded and transcribed verbatim in Bahasa Indonesia; the quotes that we reported here were translated to English (by a professional translator), while the second author, who is fluent in English and Bahasa, checked the accuracy of the translation with comparison to the original verbatim in Bahasa. An example of the verbatim transcript (in Bahasa) can be seen on the https://osf.io/vs6ke/files. Each participant received an incentive of IDR 100,000 (about $6).

### Coding and analysis

Transcripts of the interview were analyzed using thematic coding [61, 62] assisted by Nvivo 12 Plus software. In the early stages of the analysis, the researcher listened to the recorded interviews of the participants and read the interview transcripts. This was so that the researcher could become familiar with the participant data [61, 62]. After the two activities, the researcher used Nvivo 12 Plus software to group the participants’ answers to the interviewer’s questions.

For each group of answers, the researchers wrote the coding based on the understanding derived from the participants’ answers [61, 62]. To maintain the validity and reliability of the research results, the data analysis process was carried out in groups [63], which reduced bias as compared to that which would exist if one person had carried out the process (see reflexivity in Creswell and Creswell [64]). Authors 1, 3, 4, and 5 analyzed the transcript, first listening to the recording and making some notes, then reading the transcript, and making codes. Working in groups of two, they analyzed participants who were not interviewed by the coder. Each coder independently conducted the coding, and the process was later discussed in an online meeting with the partner (see interrater reliability e.g. Belur et al. [65]; Gisev et al. [66]). The discussion between the two coders aimed to reach an agreement on the coding of one participant and to save it in a separate file to create a codebook. However, in case of disagreement between the two coders, the second author helped with reviewing (transcript and codes from two coders). The discussion was focused on reflecting on the rationale behind the coding decisions, and the meeting ended with the “correct” codes agreed upon by both the two coders and the second author. The codebook was updated along with each interview being coded, as agreed upon by all four coders. This working group changed personnel after each group had finished analyzing data from five participants. Authors 2 and 6 reviewed the codebook at a certain time period, namely, at the beginning of the analysis process (after two participants’ data had been analyzed), at the middle (when 10 participants’ data had been analyzed), and at the end (after all participant data had been analyzed).

After the coding process involving the 20 participants was completed, the most recent codebook was obtained containing the results of the previous coding. The four coders discussed and created themes: codes with similar meanings were grouped under the same theme; the theme was given a name and a description that best represented all the codes it contained. The data analysis activities ended with reviews and approvals from authors 2 and 6 on the codebook containing the coding, themes, descriptions of each theme, and references from interview transcripts.

## Results

**Fig. 1:**
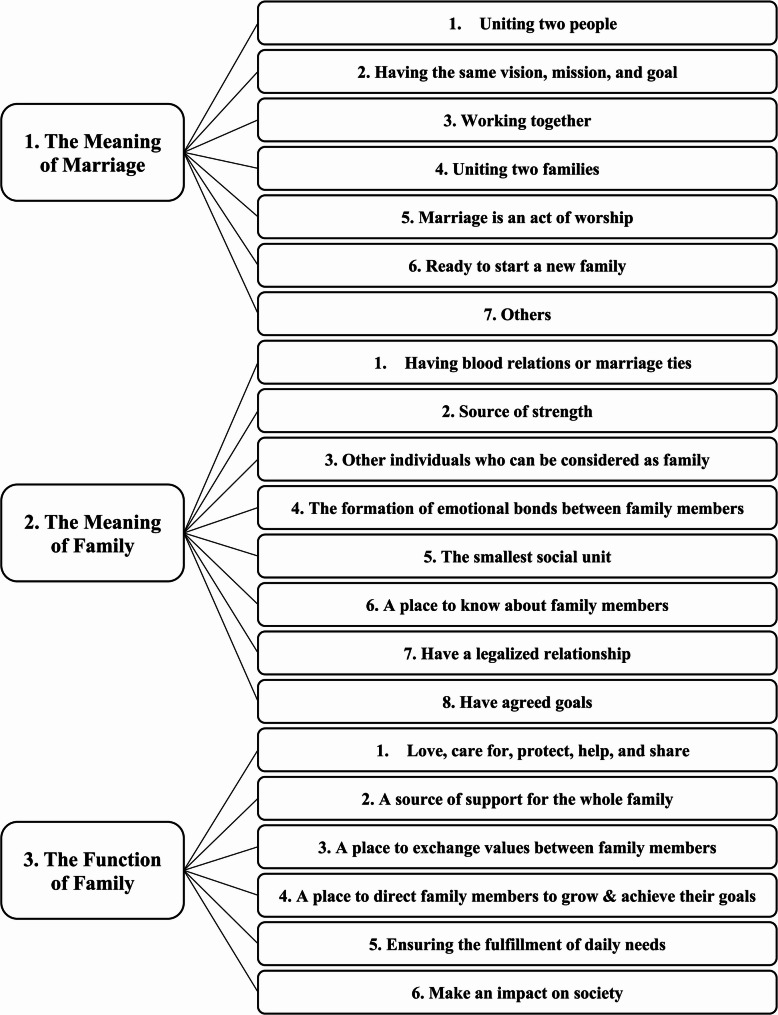
Themes of meaning of marriage, meaning of family, and function of the family

This study explored the meaning of marriage, family function, and family meaning among married people living in Indonesia. We found seven themes regarding the meaning of marriage, eight regarding the meaning of family, and six regarding the function of family (see Fig. 1). We describe in detail the themes found in the present study.

### The meaning of marriage

Participants explained the meaning of marriage based on their experience of being married. Various answers regarding the meaning of marriage were grouped into seven themes:

1. *Uniting two people.*The meaning of marriage as uniting two people was expressed by participants in different ways. Specifically, marriage unites two people; marriage brings together two different people; two people become one; and I and you become we. Examples of narratives delivered by participants are as follows:
  In my opinion, the meaning of marriage is when two people, a man and a woman, who already felt comfortable with each other and decide to unite in the smallest social unit, that’s the family, and then they are trying to build their life together. (Participant C, man, Javanese-Balinese, 30 years old, length of marriage two years, Catholic, S2/master’s degree)
  Uniting our differences; my husband and I come from very different tribes, mine is Sumbawa, he is Sumatran. In these three years I had to learn to understand, to try to make decisions together, to learn to defend each other. We live on the island of Java, away from our families, so we have to really respect each other, understand each other, and always keep arguments to a minimum. (Participant G, woman, Sumbawa, 29 years old, length of marriage three years, Islam, S2/master’s degree)
  To be married is to share with someone I love; to live a life where we take care of each other, love each other, take care of each other’s children. I’m happy in this life because I have someone who can be a friend for life. If we have a problem, for example something happens with my parents, I have a friend I can talk to, I can share everything with my partner… We also share roles in the family; sometimes I cook, he does the washing. We complement each other; we cover each other’s shortcomings… We usually manage our finances together; we discuss our income, expenses, savings, etc… (Participant B, woman, Batak, 31 years old, length of marriage five years, Catholic, undergraduate degree)

Participants A (woman, Javanese, Islam), L (woman, Sundanese, Islam), R (man, Minahasa, Protestan), S (woman, Balinese, Hindu) who have different cultural backgrounds, also mentioned the marriage as union between two people.

(2) *Having the same vision, mission, and goal. *Participants described the same vision, mission, or goals between husband and wife; having common aspects to be achieved in marriage; having the same ideas regarding how to live married life and parenting; and having similarities between husband and wife. This category also included participants' explanations of the importance of continued commitment to achieving the vision, mission, or goals of the marriage. Examples of narratives are as follows:
  The point is that marriage has the same goal. For me the goal is twofold because I am a Muslim. In my religion there is a worldly goal and an afterlife goal. For the worldly goals, as a husband and wife before marriage, we have committed to be open with each other about what we are looking for in this life. For example, I have a goal of becoming a working woman, whether my husband can provide that, can accept that, and vice versa. Personal goals have to be in line with each other; in the end we decide to go there together and support each other. Likewise with the hereafter, how to worship, we support each other in worship. (Subjek N, woman, Sundanese, 24 years old, length of marriage four months, Islam, S2/master’s degree)
  We're two people who may have different thoughts. How can we [have] one goal? We have a common goal. For example, I work out of town; we are committed, my husband allows me to work, but we don't neglect the children. When I'm out of town, my husband looks after the children at home, and vice versa when he's out of town. (Participant Q, woman, Balinese, 40 years old, length of marriage 16 years, Hindu, S2/master’s degree)
  If the couple already know each other before marriage, understand each other, trust each other, then they begin to organize the goals of the household, design what they want to do in the household; marriage is done to achieve these goals. (Participant R, man, Minahasa, 36 years old, length of marriage 10 years, Protestant, undergraduate degree)

Participants H (man, Javanese, Islam) and S (woman, Balinese, Hindu) also mentioned the similar things with N, Q, R; a marriage is defined by a shared vision and mission.

(3) *Working together.* Participants described marriage as an attempt to build a new family together; a continuous joint effort over time. In addition, participants mentioned that many events and problems can arise in a marriage. Therefore, marriage also means being ready to face these problems. Examples of narratives are as follows:
  Ready to face things. (Participant A, woman, Javanese, 33 years old, length of marriage 13 years, Islam, S2/master’s degree)
  When you get married, you make a commitment to your partner; that commitment is to live together, to raise children and to live as a family. (Participant I, woman, Javanese, 49 years old, length of marriage 21 years, Islam, S3/doctoral program)
  For my marriage, the most important thing is "each other". Mutual acceptance, mutual understanding, mutual effort so that we both feel valued. We try, the point is how we all (me and my husband, me and my extended family, my husband and my extended family) feel each other's efforts. We feel each other's efforts to make us happy, to accept us all. If even one person feels that there is no effort to be happy, negative thoughts will definitely arise. (Participant O, woman, Balinese, 25 years old, length of marriage six years, Hindu, senior high school)
(4) *Uniting two families*. Some participants interpreted marriage as the union of two families; uniting two different families; and two families becoming one. Some examples of narratives delivered by participants are as follows:
  Marriage is not only uniting two people, two individuals, two humans, two persons, but it unites two families, then unites two customs, especially those of us in Bali, the customs is very different, let alone each region, every district, each “banjar” is sometimes different in customs, so it takes a long time to be able to unite the two so that they are in harmony, so that they are harmonious, so that they can accept each other's differences. The meaning of marriage is it unites two thoughts. Then people who are part of the family are no longer part strangers, they become our family where your brother is my brother.
  Marriage is not just the two of us, there is my family, my husband's family, with different cultural customs, different religions, so we have to adapt to each other all the time. (Participant P, woman, Sundanese, 40 years old, length of marriage four years, Buddhist, S2/master’s degree)
  Uniting a couple as husband and wife, uniting two families. For example, when my father was sick, my in-laws came; they care for each other. (Participant T, woman, Javanese, 30 years old, length of marriage one year, Buddhist, associate degree).
  We were united in marriage, bringing our two families together. So this isn't just about my husband and me, but also our extended families. (Participant L, woman, 43 years old, Sundanese, length of marriage 19 years, Islam, S3/doctoral degree)
(5) *Marriage is an act of worship.* Marriage was described as a religious guideline; according to the Sunna of the Prophet (Muhammad); to perfect religion; and God's command. Examples of narratives are as follows:
  For me, marriage is also an act of worship; because the family, according to our faith (Catholic), is a place of worship, a vehicle of worship; so it is appropriate that we protect it because it is an act of worship for us. (Participant F, woman, Batak, 50 years old, length of marriage 25 years, Catholic, S2/master’s degree)
  Marriage, according to my religion (Islam), is done to perfect religion. Marriage is a sunnah of the Prophet; marriage is a once-in-a-lifetime act of worship that can last for the rest of our lives as long as we are married. (Participant M, man, Sundanese, 27 years old, length of marriage seven months, Islam, professional program)
  From a religious point of view, marriage is an act of worship. I'm Catholic, and there's a verse in the Bible that says, "Be fruitful and multiply, and fill the earth”. The purpose of marriage in our religion is to have offspring. (Participant K, man, Javanese, 34 years old, length of marriage seven years, Catholic, undergraduate degree)
(6) *Ready to start a new family*. Getting married also means starting a new family or building a new family. Examples of narratives are as follows:
  Marriage is the coming together of a man and a woman who are suited to each other, who support and respect each other, and who have the same desire to start a family. (Participant P, woman, Sundanese, 40 years old, length of marriage four years, Buddhist, S2/master’s degree)
  Procreate; continue the generation. (Participant D, woman, Batak, 30 years old, length of marriage five months, Protestant, undergraduate degree)
(7) *Others.* The participants shared several other answers, including marriage as a sign of suitability; marriage as a special relationship; and getting married means adding happiness and is a sign of maturity (achievement). Examples of narratives are as follows:
  We can formally establish a relationship that only husband and wife can enjoy, which is not possible in other relationships, such as sexual relationships. A special (relationship) unites many things, for example the financial affairs of husband and wife. My wife is my responsibility and vice versa. (Participant C, man, Javanese-Balinese, 30 years old, length of marriage two years, Catholic, S2/master’s degree)
  Getting married is maturing. It is the final peak (achievement) to be reached. (Participant E, woman, Batak, 30 years old, length of marriage two years, Protestant, undergraduate degree)

### The meaning of family

When the participants were asked about the meaning of family based on their marriage experiences, as well as on their experiences with their families of origin, almost all mentioned that family is related by blood or marriage. Participants mentioned that people can become family by blood or marriage and that what is referred to as family is a father, a mother, siblings, stepsiblings, and in-laws.

1. *Having blood relations or marriage ties*. Participants commented on becoming family by blood relation or marriage, for example, father, mother, sibling, stepbrother, mother-in-law, son-in-law, and brother-in-law. Examples of narratives are as follows:
  Family are people who are blood relatives, blood related to us, [and] then when we get married; her family becomes my family, my family becomes her family too. (Participant D, woman, Batak, 30 years old, length of marriage five months, Protestant, undergraduate degree)
  A family [consists] of a father, a mother, and children. And this father and mother are already married. (Participant E, woman, Batak, 30 years old, length of marriage two years, Protestant, undergraduate degree)
  Extended family includes cousins, uncles, aunts; nuclear family includes parents, husband, children, siblings, brothers-in-law, and sisters-in-law. (Participant G, woman, Sumbawa, 29 years old, length of marriage three years, Islam, S2/master’s degree)

Participants F, H, I, J, L, and R also mentioned blood or biological relatives when they explaining the meaning of family.

(2) *Source of strength*. Family is described as a source of strength that can provide positive energy for the participant. This energy source can also be interpreted as a source of emotional support, a place for family members to grow, a place to cultivate values for members, a place of comfort, a place to return to, and a place to ask for help.
  What is family… (It’s) where we first grew up, our sources of energy when we want to go somewhere, and also family, no matter how far we go, it’s where we come back to, the most comfortable place. (Participant A, woman, Javanese, 33 years old, length of marriage 13 years, Islam, S2/master’s degree)
  Our family is where we feel good and bad. If we have a problem, we can share it with our family. Share it with your husband or your children. Even when we’re happy, it’s better to tell our family than other people. (Participant Q, woman, Balinese, 40 years old, length of marriage 16 years, Hindu, S2/master’s degree)
  Family is where we share. Since I’ve lived away from my family since high school, my family is the place I come home to. Without us having to tell them, they know when we are in trouble and need a hug. (Participant T, woman, Javanese, 30 years old, length of marriage one year, Buddhist, associate degree)
  In my family of origin with my brothers and sisters, it was important for us to be united, to understand each other. Now I am married, the children are young, husband and wife trust and support each other. The nuclear family strengthens each member of the family. (Participant R, man, Minahasa, 36 years old, length of marriage 10 years, Protestant, undergraduate degree)

(3) *Other people who can be considered family*. Participants commented on feeling close to other individuals, feeling that they are brothers or sisters due to the existence of a level of comfort with each other, the fact that the other person understands the participant, helping each other, and listening to each other. Usually, such a person is a friend of the participant.
  Hmm, so was to me, since high school I had friends who sometimes if we couldn't speak to our family (I can speak to her), being the closest friend feels like a sister… which means that we were not related by blood but could understand us like that, for example, we could even in silence but they could know what we were thinking or feeling. (Participant J, woman, Tionghoa, 43 years old, length of marriage eight years, Catholic, undergraduate degree)
  A friend that I am very close to, that I trust and can confide in, that I feel is my family. I treat them like family; I give them more attention than my other friends. (Participant L, woman, 43 years old, Sundanese, length of marriage 19 years, Islam, S3/doctoral degree)
  Sometimes there is a psychological bond with friends, so that friends are considered family, like family. My husband and I are both migrants (from Bali to Java). Here we meet fellow Hindus or non-Hindus with whom we struggle; even though we are not related by blood, it feels like family. (Participant S, woman, Balinese, 40 years old, length of marriage 10 years, Hindu, S2/master’s degree)
(4) *The formation of emotional bonds between family members*. Participants experienced strong and close emotional bonds with their family members. They experienced love and affection for family members. Participants also described how their love enabled them to do what was best for their family. For example, there was a sense of closeness/togetherness that went beyond closeness with other people, a sense that the bond would continue to exist, and a sense of mutual protection, mutual acceptance, and mutual remembrance between family members.
  A family is a group of people, who we will feel like we belong to each other, a sense of belonging in the sense of, will we care more, be more responsible when something happens to each other. For example, if one family member has a problem, we have to help whether it's looking for a solution, or whether it's getting involved in solving the problems that occurred. So, the meaning of family is deeper and broader than that of friends or others. Someone we have to protect, it's like that of a family. (Participant L, woman, 43 years old, Sundanese, length of marriage 19 years, Islam, S3/doctoral degree)
  It's more about closeness, more intensity with family than with others. From then until now it's mostly with the family. (Participant N, woman, Sundanese, 25 years old, length of marriage 4 months, Islam, S2/master’s degree)
  My sense of emotional attachment is more to family. (Participant P, woman, Sundanese, 40 years old, length of marriage 4 years, Buddhist, S2/master’s degree)
(5) *The smallest social unit*. Participants commented on the family as the smallest unit in society; the fact that society is made up of families; and the quality of the family determines the quality of society.
  Family… the smallest part of society that has common goal. (Participant H, man, 26 years old, Javanese, length of marriage one year, Islam, professional undergraduate degree)
  Become a part of Indonesian society. (Participant F, woman, Batak, 50 years old, length of marriage 25 years, Catholic. S2/master’s degree)
  The family is a small unit in society; as part of society we have a big responsibility to contribute to society. We have to be individuals who don’t break the rules, who do things that are in line with social norms. (Participant I, woman, Javanese, 49 years old, length of marriage 21 years, Islam, S3/doctoral degree)
(6) *A place to know about family members*. Each family member has a different character, so in a relationship they will continuously learn more about and understand each other.
  So indeed the family consists of several people who, like me and my husband, are different, our children too, so we have to know each other. (Participant J, woman, Tionghoa, 43 years old, length of marriage eight years, Catholic, undergraduate degree)
(7) *Have a legalized relationship*. Family is defined as a relationship that can also be formed due to a legal process (legally recorded marriage, adoption, inheritance rights, rights and obligations of the husband, wife, and children are protected by law).
  I have two mothers and two fathers. I was adopted by my aunty. So legally, I am the child of my aunty, I call my aunty “Ibu”, but de facto I am the child of who I now call “Mama”. (Participant H, man, Javanese, 26 years old, length of marriage one year, Islam, professional program)
(8) *Have agreed goals*. Families that are defined as individuals and their partners have the same goals, vision, and mission, which were agreed upon before the decision was made to get married.
  The family… (its members) have agreed goals according to religion. (Participant H, man, Javanese, 26 years old, length of marriage one year, Islam, professional program)

### The function of family

Participants’ responses regarding the function of family varied. Some provided long, detailed answers based on their experiences not only with their nuclear family but also with their extended family, while others provided rather brief answers. The following themes emerged:

1. *Love, care, protect, help, and share*. The family functions to look after each other and protect its members from harm (physical, psychological, etc.). Participants explained that the function of family is to help its members solve problems, especially in times of difficulty; care for each other; remind each other; be a place to share; and provide unlimited and unconditional love for its members.
  Hmm… the function of the family… is to protect, I think to protect. Then the function of the family is to receive attention. To protect, to receive attention, to be loved. Then the family function is where we, each of the whole family members, can tell anything. Then we can love each other, then we get protection, we feel protected, feel loved. (Participant B, woman, Batak, 31 years old, length of marriage five years, Catholic, undergraduate degree)
  The function of the family is more like a house, where you can pour out all your heart, yes, it's a shelter and a place to share. (Participant M, man, Sundanese, 27 years old, length of marriage seven months, Islam, professional program)
  A place of refuge; a place to seek comfort, to ask questions. A place to get early experience of life… from many people (in the family) we can get all kinds of experience. When we are happy, when it is difficult, that is where we take refuge… Family is like an umbrella, rain or shine, it is a refuge. (Participant S, woman, Balinese, 40 years old, length of marriage 10 years, Hindu, S2/master’s degree)
2. *A source of support for the whole family*. Participants indicated that the function of family is to provide support for each family member. In good and bad situations, the family continues to provide support. In times of success, the family shows appreciation. In times of failure, the family provides encouragement. A family also allows its members to maintain their emotional relationships with each other. Examples of explanations given by participants are as follows:
  Family functions can be a reminder and a refreshing place, a refreshing place in the sense that we can play with them, what makes you feel comfortable, what makes you feel cozy, so when you are confused, you will definitely return to the family, tell them your story. Family functions are also a place to tell stories, a place to share happiness, a place to share sadness, a place to share strength. Also to be a source of strength for the family, to be a supporter. (Participant L, woman, Sundanese, 43 years old, length of marriage 19 years, Islam, S3/doctoral degree)
  Family functions… as a place to go home to, or a refuge, a place to share problems or happiness. When we have a problem, the first people we share it with or ask their opinion are definitely our family, the first people to give us peace of mind when we have a problem, so we cool down the atmosphere (calm our hearts), after calming our hearts, we usually ask our family for advice too. (Participant N, woman, Sunda, 25 years old, length of marriage four months, Islam, S2/master’s degree)
3. *A place to exchange values between family members*. The family is a place where members transmit values—universal life values, religious values, morals, etiquette, and manners. The transmission of these values can occur across generations in families; it can be from parent to child, from child to parent, or among other family members.
  Hmm, at some levels it might educate the mother and father as well. Like me, once I unite with my wife, my wife also often gives criticism to me like that, on the other hand I give criticism to my wife. Maybe later when my children grow up, who knows, they'll give some new insight for me. So it's not just parents to children but all parents to children, but all elements of the family; uniting two, two different family paradigms, which we finally get to an understanding of, and that's what we will teach or educate our descendants. (Participant C, man, Javanese-Balinese, 30 years old, length of marriage two years, Catholic, S2/master’s degree)
  It's the family that will definitely tell you about the customs. For us Batak who are newly married, there are many events we have to attend that we don't know about. For example, if a relative is getting married, what should we bring? They will tell you, for example, from the women's side, you bring this, the men bring that, so we know more about the custom, we talk about it. There are many Batak clans that are related to each other. (Participant D, woman, Batak, 30 years old, length of marriage five months, Protestant, undergraduate degree)
  The family becomes a place of exchange of views and values that we will later teach our children; … by bringing together two different family paradigms, we finally come to an understanding, and that is what we will teach our children. (Participant E, woman, Batak, 30 years old, length of marriage two years, Protestant, undergraduate degree)
4. *A place to direct family members to grow and achieve their goals*. Families, especially parents, direct their children to achieve their life goals. Before making a certain decision or taking a certain action or step, participants usually ask for or listen to the opinions of family members. This is regarded as helping family members develop themselves, grow to be better, have advantages, or form their identity.
  The place where we grow. (Participant A, woman, Javanese, 33 years old, length of marriage three years, Islam, S2/master’s degree)
  The function of the family for its members is actually more of a director. What do our directors want us to be, with what way to get there, also as I mentioned earlier, a place to go home, or a place of refuge where we can share our problems, or when we have something wrong. There is a certain happiness like that. Then what is the function of the family, actually, it was already represented by the director. The director includes education, then includes character education, like manners, ethics, all kinds of things already in the director. (Participant N, woman, Sunda, 25 years old, length of marriage four months, Islam, S2/master’s degree)
  … our focus is on the children, preparing them for the future. (Participant K, man, Javanese, 34 years old, length of marriage seven years, Catholic, undergraduate degree)
5. *Ensuring the fulfillment of daily needs*. Families can meet the daily needs of their members, including food, clothing, housing, health needs, education, etc.
  … support each other from the material side maybe. Even though my husband once said I actually don't have any obligation to work, but if I can empower myself well, I can do self-actualization, and produce something, then why don't we support each other. There are times when I am helped and there are times when I also help, we help each other. (Participant A, woman, Javanese, 33 years old, length of marriage three years, Islam, S2/master’s degree)
6. *Make an impact on society*. The family as the smallest unit of society is considered to have the function of impacting its surroundings.
  The family function is as a means to go to religion, but it also has something to do with the state… for the state, because our status in the state is the smallest structure in society, so whether we like it or not, we have the least influence on society. (Participant H, man, 26 years old, Javanese, length of marriage one year, Islam, professional program)

## Discussion

The present study aimed to explore how married Indonesian participants perceive the meaning of marriage, the meaning of family, and the function of family based on their experiences. We found seven themes for the meaning of marriage (e.g., uniting two people; having the same vision, mission, or goal; etc.), eight themes for the meaning of family (e.g., having blood or marital ties; source of stress; etc.), and six themes for the function of family (e.g. loving, caring, protecting, helping, and sharing; a source of support for the whole family).

### The meaning of marriage

In the present study, in a diverse sample of ethnic background, religion, age, education, and length of marriage, the majority of participants interpreted marriage as the union of two individuals and two different families. In this process of becoming one, husband and wife get to know each other, work together, share, communicate with each other, complement each other, understand each other, meet each other’s needs, support each other, and accept each other – as our participants indicated. This finding is in line with the views of other researchers, for example, Olson et al. [67] and Kusuma, Putri, and Pratisti [37]. The previous studies showed that marriages are perceived as an emotional and legal commitment of two people to share emotional and physical intimacy, various tasks, economic resources, and values. Moreover, the current qualitative findings are strongly in line with findings based on self-expansion theory, showing that partners become part of each other’s self-concept (i.e., including the other in the self [68]). Broderick [69] discussed the notion that marriage is the joining of two families and social networks. When individuals marry, they marry not only each other but also their partner’s family and friends. Married life is characterized by mutual communication, mutual support, sharing, and mutual cooperation, which is also in line with the findings of previous research in several countries; this became known as the International Family Strength Framework [67, 70, 71]. According to Coontz [72], marriage today is more fair and fulfilling for couples and their children than ever before.

Marriage is also perceived as a relationship in which husband and wife have the same vision, mission, and goals. This finding is in line with previous research [73–75]. Having a similar goal in marriage—what individuals want to achieve in marriage—is one of the determinants of marital satisfaction [76, 77], and the purpose of marriage can vary across the life span, depending on life and cultural transitions. In today’s society, married couples have higher expectations regarding their marriage and must invest more time, effort, and other resources, to increase their marital satisfaction [78]. When couples have a positive and optimistic view of achieving higher expectations, these expectations motivate more constructive behavior toward marital challenges, thus preventing a decline in marital satisfaction over time [74, 75].

Participants also mentioned that having shared marriage goals can encourage partners to commit in the relationship. Having a purpose in marriage helps couples agree on things that must be done to maintain the marriage. This commitment is a promise between partners that must be kept continuously. Commitment is one of the characteristics of a stable marriage and family [67, 71, 79]. In ongoing relationships, the perception that both partners are equally participating emerges as the prime factor for promoting commitment [80]. Increasing commitment to each other is correlated with fewer marital problems and increasing expressions of affection (love) between partners [81].

A small number of participants interpreted marriage as an act of worship, carried out because of religious guidance (in particular the Islamic and Catholic participants). This finding is consistent with previous studies reporting on the belief that marriage should be made according to religious rules [82, 83]. For Muslims, marriage is carried out as a form of obedience to God’s commands and to imitate the behavior of the prophet Muhammad; married life (family life) and its general procedures are regulated in the Quran. More generally, for religious groups, marriage is a sacred union that is “something more”—more than the self, more than the couple, and more than the family unit [84]. Indonesia is a country that attaches great importance to religion in the lives of its people, including regulating the place of religion and God in married life [30, 85]. Therefore, it is not surprising that participants in this study reported that they adhered to these religious values in their married lives.

Previous research has shown that Indonesians in general tend to marry members of the same ethnicity [86] despite being accustomed to ethnic and religious differences (e.g., Parker et al. [87]). Given that we found quite some overlap in perspectives on the meaning of marriage in our diverse sample, the broader challenges that participants experience and face in their marriages may be similar for participants from diverse backgrounds as their marriages are embedded in the broader Indonesian culture. For example, participants of various backgrounds may face similar Indonesian norms about marriage (e.g. marriage includes being part of an extended family), and they need to adapt to similar external challenges (e.g. financial strain). Such similar challenges may be associated with similar perspectives on the meaning of marriage. Our findings suggest that ideas and beliefs about marriage are largely shared, irrespective of cultural, ethnic, or religious background.

In general, the meaning of marriage mentioned by the participants in this study is similar to previous studies conducted both in Indonesia and in Western cultures. However, participants’ belief that marriage is an act of worship is quite unique. Marriage is indeed associated with God in Indonesian culture and law, as stated in the Indonesian Marriage Law. As such, in Indonesia the place of religion and God is formally regulated in married life [30, 85]. This uniqueness is also relevant to the fact that religious institutions are responsible for organizing marriage preparation in Indonesia (for example, the KUA for Muslims and the Church for Christians and Catholics).

### The meaning of family

Concerning the meaning of family, participants believed that families are characterized by blood relations or formed because of marital relations. This also establishes a legal relation by law, with each member having rights and responsibilities protected by law. This is in line with the views of experts and findings in other surveys (see Mahoney [88], Olson et al. [67], Struthers & Bokemeier [89], Turtiainen et al. [90], Amendment to Law No. 23 of 2002 on child protection [91], Wiratri [92]). Generally, families consist of a husband and a wife with or without children, or a father and children, or a mother and children, with or without other family members.

The meaning of family is also seen as an affiliation that provides psychological resources. Participants mentioned that family was perceived as a source of emotional support, the most comfortable place for all family members, of sharing love and affection, and a place where members can learn to understand each other; these experiences are consistent with previous studies [90, 93, 94]. Also similar to previous findings [67, 90], some participants shared that families involve two or more individuals who are committed and share intimacy, resources, decision-making responsibilities, and values. The family is the main source of support for its members, including in critical situations such as illness [95]. When the family grows, there is also increasing love, affection, and attention, as well as increasing responsibility to care for all family members.

Family is not always a biological relationship. In this study, some participants mentioned that they considered their best friends to be family. Participants expressed the meaning of family as the feelings of closeness, comfort, understanding, and listening to each other experienced by participants and their best friends who are not biologically related. Family can be defined as people who care for us, support us, and feel like family to us but who are not related by blood or law [96]. The literature also mentions that the Javanese like to create new family networks by treating unrelated people as family members (Geertz, 1961 in Subandi [97]). Today, the Indonesian community defines family more broadly, not limiting it to roles (husband, wife, children), location (must live under one roof), or the consideration of blood, marriage, or adoption [92]. Participants mentioned family as an emotional bond between each of its members. This is consistent with previous studies that consider Indonesia as a collectivist society in which its members have an interdependent self-construal belief, with the view of the self as inherently connected to others. This sense of connection (emotional bonding in this study) is associated with positive feeling and well-being [98]. Participants also viewed a family as the smallest unit in society and indicated that it determines the quality of society. As far as we know, this was not found in previous studies.

As with the meaning of marriage, participants from different backgrounds (i.e., religion, education, ethnicity) shared a similar meaning of family. Family is central to the lives of many ethnic groups living in Indonesia and is a source of support (e.g., Mangundjaya [99] and Subandi [97]). For example, previous research showed that in Javanese society (Geertz, 1961 in Subandi [97]), the family is considered to provide a sense of community *tentrem* (peace), *hangat* (warmth), and *kasih sayang* (unconditional love); the family is also a source of affection and instrumental help (see French et al. [100]. Other research showed that among the Sundanese, Javanese, Balinese, and Batak, family members are taught to love and respect their elders, there should be mutual respect and tolerance between family members, there should be mutual care between husband and wife and the extended family, and loving each other and helping are important family values (e.g., Adnyani & Suwastini [101], Harianja & Sudrajat [102], Muda & Suharyanto [103], Smith-Hefner [33]). While such previous research has often focused on specific populations in Indonesia with a specific cultural background (e.g., Sundanese, Javanese, etc.), the current findings indicate that their perspectives on the meaning of family might be quite similar.

The findings on the meaning of family in this study support the findings of previous studies, but the participants’ perception that other people (i.e. not blood-related) can be family and that family is the smallest social unit seems quite unique. Indonesia is known as a nation with collectivistic values. In a collectivistic culture, self-evaluation is closely linked to the evaluation of others (group, family, society), there is “we” awareness, with a norm that harmony should always be maintained, relationships prevails over task performance, and others are often classified as in-group or out-group (see Hofstede [104]). Thus, social harmony, being a part of a group, and contributing to the group (society) are important values in Indonesian families.

### The function of family

Regarding the functions of a family, participants mentioned the following: to look after, protect, and provide support to each other; to help solve problems; to care for and remind each other; to serve as a place for the sharing of unlimited affection; and to meet the daily needs of its members. These functions support the findings of previous studies (see Harden et al. [93] Mahoney [88], Patterson [51]). A well-functioning family will affect children’s growth (see Duijster et al. [105], Guenther et al. [106]).

We found that the family functions as a place where members can grow and achieve their goals in life. Parents (including older family members) are believed to be able to provide direction to other family members, for example, by offering their views before a decision is made. These findings indicate that family members are interdependent and that the personal choices of each family member are ultimately known and approved of by other family members, especially parents. Another finding was the participants’ assessment that the family must be able to have a positive impact on society. The family functions mentioned by the participants in this study are consistent with their explanation of the meaning of family. Because their family has these functions, it can be called a family, or vice versa.

### Limitations and future directions

There are several limitations to this study. First, due to the Covid-19 pandemic, data collection was conducted online using Google Meet. Older married people (e.g. more than 50 years) unfamiliar with the technology were unable participate in this study, although their experiences could have enriched the results of this study. The researchers found it difficult to conduct a discussion process to explore participants’ responses, including understanding the emotions that accompanied the experiences; in addition, signal limitations sometimes interfered with the communication process. Second, there were more female participants in this study; thus, future studies must be conducted to obtain a better understanding of males’ perspectives on marriage and family. More generally, while the study was meant to explore views on marriage in a diverse sample with different backgrounds, the sample size was too limited to examine systematic differences between the different cultural and religious backgrounds – a larger sample that includes more individuals from each background would allow this, and is an important step for future research. Despite the general similarities that we found in the diverse sample, there might be differences between individuals from different backgrounds that we did not study at this time. Third, we collected data from individuals rather than couples.

The current findings – showing that a diverse sample of Indonesians share quite similar views on marriage and family – form a springboard to further examine this topic in future research. For example, it would be interesting to examine more systematically potential differences between individuals from different religious, ethnic, and cultural backgrounds, that were not uncovered in the current research. Moreover, including data from both husbands and wives would provide insight into possible gender differences in the meaning of marriage. Such questions could be explored with larger samples in qualitative studies or examined through more quantitative research. For example, future quantitative research could explore how the views of marriage and family, and the functions of family as were indicated in our sample, would relate to variables like marital coping stress, happiness, and marital satisfaction.

## Conclusion

This study provides an in-depth understanding of how married people in Indonesia perceive the meaning of marriage and family. Although the participants came from different cultural backgrounds (ethnicity, religion, educational background, etc.), their perspectives on the meaning of marriage and the meaning and function of family were more or less the same. Thus, the current findings suggest that Indonesians might have a fairly consistent and shared view of the meaning of marriage and family. The current findings provide input for further future explorations into similarities as well as potential differences across Indonesian cultures.

## Supplementary Information

Supplementary Material 1.

## Data Availability

The datasets used and/or analysed during the current study are available from the corresponding author on reasonable request. The example of verbatim transcript (in Bahasa) can see on the https://osf.io/vs6ke/files.

## References

[1] McGoldrick M, Preto NG, Carter B. The expanding family life cycle: Individul, family, and social perspectives. 5th ed. Boston: Pearson; 2016.

[2] Garcia-Hombrados J, Özcan B. Age at marriage and marital stability: evidence from China. Rev Econ Househ. 2024;22(1):297–328.

[3] Lee GR, Payne KK. Changing marriage patterns since 1970: what’s going on, and why? J Comp Fam Stud. 2010;41(4):537–55.

[4] Saardchom N, Lemaire J. Causes of increasing ages at marriage: an international regression study. Marriage Fam Rev. 2005;37(3):73–97.

[5] Berger P, Kellner H. Marriage and the construction of reality: an exercise in the microsociology of knowledge. Diogenes. 1964;12(46):1–24.

[6] Cherlin AJ. Degrees of change: an assessment of the deinstitutionalization of marriage thesis. J Marriage Family. 2020;82(1):62–80.

[7] Katz M. Agreement on connotative meaning in marriage. Fam Process. 1965;4(1):64–74.

[8] Timmer SG, Orbuch TL. The links between premarital parenthood, meanings of marriage, and marital outcomes. Fam Relat. 2001;50(2):178–85.

[9] Carrère S, Buehlman KT, Gottman JM, Coan JA, Ruckstuhl L. Predicting marital stability and divorce in newlywed couples. J Fam Psychol. 2000;14(1):42–58. 10740681 10.1037//0893-3200.14.1.42

[10] Berrington A, Perelli-Harris B, Trevena P. Commitment and the changing sequence of cohabitation, childbearing, and marriage: insights from qualitative research in the UK. Demogr Res. 2015;33:327–62.

[11] Hall SS. Marital meaning: exploring young adults’ belief systems about marriage. J Fam Issues. 2006;27(10):1437–58.

[12] Adegboyega LO. Influence of social media on marital stability of married adults in Ilorin Metropolis. In: Igboin BO, Adeusi So, editors. Families in Nigeria: Understanding their diversity, adaptability, and strengths (contemporary perspectives in family research). Bingley: Emerald Publishing Limited; 2022. p. 55–68.

[13] Lissitsa S. Percieved optimal marriage age in the internet era—Findings of a nationwide survey. Marriage Fam Rev. 2019;55(2):126–51.

[14] Rahman TA, Sutjipto VW, Putri KYS. The impact of exposure to online mass media on people’s attitudes in marriage decisions in Indonesia. Momentum Matrix: Int J Communication Tourism Social Economic Trends. 2024;1(4):12–23.

[15] Republic of Indonesia. Law No. 1 of 1974 on Marriage. Jakarta: Government of the Republic of Indonesia; 1974.

[16] Kemenpppa BPS, Profil perempuan. Indonesia 2019 [Profile of Indonesian women 2019]. Kementerian Pemberdayaan Perempuan dan Perlindungan Anak; 2019. Available from: https://www.kemenpppa.go.id/lib/uploads/list/b4bdc-profil-perempuan-indonesial-_2019.pdf.

[17] Utomo A, Ananta A, Setyonaluri D, Aryaputra C. A second demographic transition in indonesia? China Popul Dev Stud. 2022;6(3):288–315. 36313816 10.1007/s42379-022-00115-y PMC9589626

[18] BPS - Statistics Indonesia. Population 15 years of age and over by age group and type of activity during the previous week, 2008–2024. BPS - Statistics Indonesia. 2024. Available from: https://www.bps.go.id/en/statistics-table/1/MTkwNCMx/penduduk-berumur-15-tahun-ke-atas-menurut-golongan-umur-dan-jenis-kegiatan-selama-seminggu-yang-lalu--2008---2024.html.

[19] Carroll JS, Badger S, Willoughby BJ, Nelson LJ, Madsen SD. McNamara Barry C. Ready or not? Criteria for marriage readiness among emerging adults. J Adolesc Res. 2009;24(3):349–75.

[20] Novianti LE, Purba FD, Noer AH, Kendhawati L. Pernikahan Dalam perspektif Masyarakat Bandung [Marriage in the perspective of Bandung people]. J Psikogenesis. 2018;6(1):79–90.

[21] Mawaddah S, Safrina L, Mawarpuri M, Faradina S. Perbedaan Kesiapan Menikah Pada Dewasa Awal Ditinjau Dari Jenis Kelamin Di Banda Aceh [The Difference of marital readiness on emerging adult based on gender in Banda Aceh]. Jurnal EMPATI. 2019;8(1):320–8.

[22] Ningrum DNF, Latifah M, Krisnatuti D. Marital readiness: exploring the key factors among university students. Humanitas Indonesian Psychol J. 2021;18(1):65–74.

[23] Rahmah N, Kurniawati W. Relationship between marriage readiness and pregnancy planning among prospective brides. J Public Health Res. 2021;10(1suppl):2405. 10.4081/jphr.2021.2405 PMC9309681 34060752

[24] Indonesia. go.id. Suku bangsa [Ethnic groups]. Indonesia.go.id. 2017. Available from: https://indonesia.go.id/profil/suku-bangsa/kebudayaan/suku-bangsa.

[25] Aziz EA. Bahasa daerah dalam impitan zaman [Regional languages under the pressure of time]. Badan Pengembangan dan Pembinaan Bahasa: Kementerian Pendidikan, Kebudayaan, Riset dan Teknologi. 2023. Available from: https://badanbahasa.kemdikbud.go.id/artikel-detail/3848/bahasa-daerah-dalam-impitan-zaman.

[26] BRIN. Keragaman agama dan kepercayaan di Indonesia dapat dimasukan dalam Ensiklopedi nasional [Religious and belief diversity in Indonesia can be included in the national encyclopedia]. Organisasi Riset Ilmu Sosial dan Humaniora: Badan Riset dan Inovasi Nasional. 2022. Available from: https://ipsh.brin.go.id/2022/06/08/keragaman-agama-dan-kepercayaan-di-indonesia-dapat-dimasukan-dalam-ensiklopedi-nasional/.

[27] Afnan D. Mitos larangan menikah antara orang Jawa dengan orang Sunda dalam perspektif masyarakat modern [Myth of the marriage ban between Javanese and Sundanese people from a modern societal perspective]. Arif: Jurnal Sastra dan Kearifan Lokal. 2022;2(1):157–76.

[28] Beydha I. Conflict communication in marriage Indonesia Minangkabau matrilineals’ culture women with Malay patrilineals’ culture men in Medan, Indonesia [Doctoral dissertation]. [Selangor]: Universiti Putra Malaysia; 2007. Available from: http://psasir.upm.edu.my/id/eprint/5428/1/IPSS_2007_1.pdf.

[29] Manggola A. Pola komunikasi Antarbudaya Pasangan suami-istri Beda suku: Antara Suku Pekal Dengan Suku Jawa Di Bengkulu Utara [Intercultural communication patterns of interethnic married couples: between the Pekal and Javanese ethnic groups in North Bengkulu]. JOPPAS: J Public Policy Adm Silampari. 2021;3(1):26–39.

[30] Seo M. Falling in love and changing Gods. Indones Malay World. 2013;41(119):76–96.

[31] Yunita K, Setyari EP, Safitri F. Cultural identity negotiation as a form of conflict management: A study of intercultural communication strategies in Batak-Chinese marriage. Int J Multicultural Multireligious Underst. 2022;9(1):717–23.

[32] Dhofier Z. Kinship and marriage among the Javanese Kyai. Indonesia. 1980;29:47.

[33] Smith-Hefner NJ. The new Muslim romance: changing patterns of courtship and marriage among educated Javanese youth. J Southeast Asian Stud. 2005;36(3):441–59.

[34] Sari Y, Afiatin T, Subandi S, Setiawan HW. Sundanese family strength: A preliminary study. Jurnal Ilmiah Peuradeun. 2020;8(3):567.

[35] Sitompul R, Alesyanti, Ridwan M. Domestic violence as initiated by Batak culture in East Medan, Indonesia. J Hum Behav Soc Environ. 2020;30(7):835–42.

[36] Nurviana A, Hendriani W. Makna Pernikahan Pada generasi milenial Yang Menunda Pernikahan Dan Memutuskan Untuk Tidak Menikah [The meaning of marriage for millennials who postpone marriage and choose not to marry]. Buletin Riset Psikologi Dan Kesehatan Mental (BRPKM). 2021;1(2):1037–45.

[37] Kusuma Putri I, Pratisti WD. Makna Pernikahan Bagi Wanita Yang Menikah Kembali [The meaning of marriage for women who remarry] [Undergraduate theses]. [Surakarta]: Universitas Muhammadiyah Surakarta; 2018.

[38] Permatasari AL. Dibalik Pernikahan Dini: studi Tentang Makna Pernikahan Bagi Pasangan Menikah Dini Di purwokerto Kabupaten Banyumas [Behind early marriage: A study on the meaning of marriage for early married couples in purwokerto, Banyumas Regency] [Undergraduate theses]. [Purwokerto]: Universitas Jenderal Soedirman; 2018.

[39] Prabowo GA. Konstruksi sosial Tentang Perkawinan Disabilitas Tunanetra Di surabaya: studi deskriptif Tentang Makna Perkawinan Bagi Wanita normal Yang Menikah Dengan Disabilitas Tunanetra Anggota PERTUNI [Social construction of marriage with visual impairment in surabaya: A descriptive study on the meaning of marriage for non-disabled women married to visually impaired individuals who are members of PERTUNI] [Undergraduate theses]. [Surabaya]: Universitas Airlangga; 2014.

[40] Febrianti E. Makna perkawinan bagi pasangan menikah usia muda di Desa Tapa Baru Kecamatan Sikap Dalam Sumatera Selatan [The meaning of marriage for young married couples in Tapa Baru Village, Sikap Dalam Subdistrict, South Sumatra] [Thesis]. [Padang]: Universitas Negeri Padang; 2013.

[41] Himawan KK, Bambling M, Edirippulige S. Singleness, religiosity, and the implications for counselors: the Indonesian case. Eur J Psychol. 2018;14(2):485–97. 30008958 10.5964/ejop.v14i2.1530 PMC6016032

[42] Rahmalia D, Sary N. Makna pernikahan pada istri yang menggugat cerai suami [The meaning of marriage for wives who file for divorce]. In: Prosiding Seminar Nasional Darmajaya. 2017;83–100. Available from: https://jurnal.darmajaya.ac.id/index.php/PSND/article/view/828. [cited 2022 Oct 23].

[43] Goff JM, Le, Ryser VA. Meaning of marriage for men during their transition to fatherhood: the Swiss context. Marriage Fam Rev. 2010;46(1–2):107–25.

[44] Özyiğit MK. The meaning of marriage according to university students: A phenomenological study. Educational Sciences: Theory Pract. 2017;17(2):679–711.

[45] Curran MA, Utley EA, Muraco JA. An exploratory study of the meaning of marriage for African Americans. Marriage Fam Rev. 2010;46(5):346–65.

[46] Hurt TR. Toward a deeper Understanding of the meaning of marriage among black men. J Fam Issues. 2013;34(7):859–84.

[47] Duvall ERM, Miller BC. Marriage and family development. New York: Harper & Row; 1985.

[48] Cox FD, Demmitt K. Human intimacy, relationships, marriage, and the family. In: Human intimacy: Marriage, the family, and its meaning. 11th ed. Boston: Cengage Learning; 2014. p. 29.

[49] Setyawan I, Hidayati FNR. Keluarga di mata remaja milenial: Studi deskriptif tentang makna keluarga [Family in the view of millennial teens: A descriptive study on the meaning of family]. In: Prosiding Seminar Nasional Fakultas Psikologi Universitas Diponegoro. 2021. p. 308–316. Available from: https://www.researchgate.net/profile/Imam-Setyawan/publication/353922693_Keluarga_di_Mata_Remaja_Milenial_Studi_Deskriptif_tentang_Makna_Keluarga_httpssemnaspsikologiundipacidprosiding-2021/links/611a37840c2bfa282a49e967/Keluarga-di-Mata-Remaja-Milenial-Studi-Deskriptif-tentang-Makna-Keluarga-https-semnaspsikologiundipacid-prosiding-2021.pdf.

[50] Endrawan N. Makna keluarga bagi remaja korban perceraian: Studi kasus di Kelurahan Sudiang Kecamatan Biringkanaya [Meaning of family for teenage divorce victims: Case study in Kelurahan Sudiang, Kecamatan of Biringkanaya] [Thesis]. Makassar: Universitas Negeri Makassar; 2019. Available from: https://eprints.unm.ac.id/12218/1/jurnal.pdf.

[51] Patterson JM. Integrating family resilience and family stress theory. J Marriage Family. 2002;64(2):349–60.

[52] Aswarna WE. Perubahan fungsi keluarga di kalangan keluarga orang tua tunggal [Changes in family function among single-parent families] [Thesis]. Universitas Gadjah Mada; 2006.

[53] Herawati T, Pranaji DK, Pujihasvuty R, Latifah EW. Faktor-faktor Yang Memengaruhi Pelaksanaan Fungsi Keluarga Di Indonesia [Factors affecting the implementation of family functions in Indonesia]. Jurnal Ilmu Keluarga Dan Konsumen. 2020;13(3):213–27.

[54] Anggraini MT. Perbedaan fungsi keluarga dan kualitas hidup antara mahasiswa kedokteran dan non kedokteran [Difference in family function and quality of life between medical and non-medical students] [Thesis]. Surakarta: Universitas Sebelas Maret; 2014. Available from: https://digilib.uns.ac.id/dokumen/download/41186/MTM1NzIx/Perbedaan-Fungsi-Keluarga-dan-Kualitas-Hidup-antara-Mahasiswa-Kedokteran-dan-Non-Kedokteran-TESIS-FULLTEXT.pdf.

[55] Oktowaty S, Setiawati EP, Arisanti N. Hubungan fungsi keluarga deng an kualitas hidup pasien penyakit kronis degeneratif di fasilitas kesehatan tingkat pertama [The relationship between family function and quality of life of chronic degeneratif patients in primary health care service]. Jurnal Sistem Kesehatan. 2018;4(1):1–6.

[56] Putri ARW, Permana I. Hubungan Antara Fungsi Keluarga Dengan Kualitas Hidup Lansia Di kelurahan Wirobrajan Yogyakarta [The relationship between families function and quality of life among elderly in Wirobrajan subdistrict of Yogyakarta]. Mutiara Medika. 2011;11(1):1–7.

[57] Sutikno E. Hubungan fungsi keluarga dengan kualitas hidup lansia [The relationship between family function and the quality of life in the elderly] [Thesis]. Surakarta: Universitas Sebelas Maret; 2011. Available from: https://digilib.uns.ac.id/dokumen/download/19318/NDMzMTk=/Hubungan-Fungsi-Keluarga-dengan-Kualitas-Hidup-Lansia-Microsoft-Word---FULLTEXT-TESIS.pdf.

[58] Andreu P, Dargent A, Large A, Meunier-Beillard N, Vinault S, Leiva-Rojas U, et al. Impact of a stay in the intensive care unit on the Preparation of advance directives: Descriptive, exploratory, qualitative study. Anaesth Crit Care Pain Med. 2018;37(2):113–9. 28826983 10.1016/j.accpm.2017.05.007

[59] Iravani M, Janghorbani M, Zarean E, Bahrami M. Barriers to implementing evidence-based intrapartum care: A descriptive exploratory qualitative study. Iran Red Crescent Med J. 2016;18(2):e21471. 10.5812/ircmj.21471 PMC4863155 27175303

[60] Campbell S, Greenwood M, Prior S, Shearer T, Walkem K, Young S, et al. Purposive sampling: complex or simple? Research case examples. J Res Nurs. 2020;25(8):652–61. 34394687 10.1177/1744987120927206 PMC7932468

[61] Braun V, Clarke V. Thematic analysis. In: Cooper H, Camic PM, Long DL, Panter AT, Rindskopf D, Sher KJ, editors. APA handbook of research methods in psychology: Research designs: Quantitative, qualitative, neuropsychological, and biological. Washington: American Psychological Association; 2012. p. 57–71.

[62] Braun V, Clarke V. Using thematic analysis in psychology. Qual Res Psychol. 2006;3(2):77–101.

[63] Hill CE, Knox S, Thompson BJ, Williams EN, Hess SA, Ladany N. Consensual qualitative research: an update. J Couns Psychol. 2005;52(2):196–205.

[64] Creswell JW, Creswell DJ. Research design: Qualitative, quantitative, and mixed methods approaches. 5th ed. Los Angeles: SAGE Publications Inc.; 2018.

[65] Belur J, Tompson L, Thornton A, Simon M. Interrater reliability in systematic review methodology: exploring variation in coder decision-making. Sociol Methods Res. 2021;50(2):837–65.

[66] Gisev N, Bell JS, Chen TF. Interrater agreement and interrater reliability: key concepts, approaches, and applications. Res Social Administrative Pharm. 2013;9(3):330–8. 10.1016/j.sapharm.2012.04.004 22695215

[67] Olson D, DeFrain J, Skogrand L. Marriages and families: Intimacy, diversity, and strength. 9th ed. New York: McGraw-Hill; 2019.

[68] Aron A, Lewandowski G, Branand B, Mashek D, Aron E. Self-expansion motivation and inclusion of others in self: an updated review. J Soc Pers Relat. 2022;39(12):3821–52.

[69] Broderick CB. Understanding family process: Basics of family systems theory. Thousand Oaks: Sage Publication; 1993.

[70] Defrain J, Defrain N, Lepard J. Family strengths and challenges in the South Pacific: An exploratory study in Fiji. Int J Sociol Fam. 1994;24(2):25–47. Available from: https://www.jstor.org/stable/23028651.

[71] DeFrain J, Asay SM. Strong families around the world: an introduction to the family strengths perspective. Marriage Fam Rev. 2007;41(1–2):1–10.

[72] Coontz S. Marriage: A history. New York: Viking; 2005.

[73] Kaplan M, Maddux JE. Goals and marital satisfaction: perceived support for personal goals and collective efficacy for collective goals. J Soc Clin Psychol. 2002;21(2):157–64.

[74] McNulty JK, Karney BR. Positive expectations in the early years of marriage: should couples expect the best or Brace for the worst? J Pers Soc Psychol. 2004;86(5):729–43. 15161397 10.1037/0022-3514.86.5.729

[75] Neff LA, Geers AL. Optimistic expectations in early marriage: A resource or vulnerability for adaptive relationship functioning? J Pers Soc Psychol. 2013;105(1):38–60. 23713697 10.1037/a0032600

[76] Li T, Tsang VHL, Fung HH, Qiu XL, Wang WC. Measuring dynamic goals for marriage: development and validation of the marital goal scale using Rasch modeling. Psychol Assess. 2020;32(3):211–26. 31647255 10.1037/pas0000779

[77] Li T, Fung HH. The dynamic goal theory of marital satisfaction. Rev Gen Psychol. 2011;15(3):246–54.

[78] Neff LA, Morgan TA. The rising expectations of marriage: what we do and do not know. Psychol Inq. 2014;25(1):95–100.

[79] Surra CA, Hughes DK, Jacquet SE. The development of commitment to marriage. Handbook of interpersonal commitment and relationship stability. Boston, MA: Springer US; 1999. pp. 125–48.

[80] Sabatelli RM, Cecil-Pigo EF. Relational interdependence and commitment in marriage. J Marriage Fam. 1985;47(4):931.

[81] Swensen CH, Trahaug G. Commitment and the long-term marriage relationship. J Marriage Fam. 1985;47(4):939.

[82] Rozario S. Islamic marriage: A Haven in an uncertain world. Cult Relig. 2012;13(2):159–75.

[83] Van Niekerk J, Verkuyten M. Interfaith marriage attitudes in Muslim majority countries: A multilevel approach. Int J Psychol Relig. 2018;28(4):257–70.

[84] Dollahite DC, Hawkins AJ, Parr MR. Something more: the meanings of marriage for religious couples in America. Marriage Fam Rev. 2012;48(4):339–62.

[85] Seo M. Defining ‘religious’ in indonesia: toward neither an Islamic nor a secular state. Citizensh Stud. 2012;16(8):1045–58.

[86] Utomo A, McDonald P. Who marries whom? Ethnicity and marriage pairing patterns in Indonesia. Asian Popul Stud. 2016;12(1):28–49.

[87] Parker L, Hoon CY, Raihani. Young people’s attitudes towards inter-ethnic and inter-religious socializing, courtship and marriage in Indonesia. South East Asia Res. 2014;22(4):467–86.

[88] Mahoney A. Religion in families, 1999–2009: A relational spirituality framework. J Marriage Family. 2010;72(4):805–27. 10.1111/j.1741-3737.2010.00732.x PMC3219420 22102761

[89] Struthers CB, Bokemeier JL. Myths and realities of Raising children and creating family life in a rural County. J Fam Issues. 2000;21(1):17–46.

[90] Turtiainen P, Karvonen S, Rahkonen O. All in the family? The structure and meaning of family life among young people. J Youth Stud. 2007;10(4):477–93.

[91] Republic of Indonesia. Law No. 35 of 2014: Amendment to Law No. 23 of 2002 on Child Protection. Jakarta: Government of the Republic of Indonesia; 2014.

[92] Wiratri A. Menilik Ulang arti Keluarga Pada Masyarakat Indonesia [Revisiting the concept of family in Indonesian society]. Jurnal Kependudukan Indonesia. 2018;13(1):15–26.

[93] Harden J, Backett-Milburn K, MacLean A, Cunningham-Burley S, Jamieson L. Home and away: constructing family and childhood in the context of working parenthood. Child Geogr. 2013;11(3):298–310.

[94] Meier JA, McNaughton-Cassill M, Lynch M. The management of household and childcare tasks and relationship satisfaction in dual-earner families. Marriage Fam Rev. 2006;40(2–3):61–88.

[95] Olsen KD, Dysvik E, Hansen BS. The meaning of family members’ presence during intensive care stay: A qualitative study. Intensive Crit Care Nurs. 2009;25(4):190–8. 19497746 10.1016/j.iccn.2009.04.004

[96] Braithwaite DO, Bach BW, Baxter LA, DiVerniero R, Hammonds JR, Hosek AM, et al. Constructing family: A typology of voluntary kin. J Soc Pers Relat. 2010;27(3):388–407.

[97] Subandi MA. Family expressed emotion in a Javanese cultural context. Cult Med Psychiatry. 2011;35(3):331–46. 21773874 10.1007/s11013-011-9220-4

[98] Friedman MD. Adult attachment and self-construal: A cross-cultural analysis [Doctoral dissertation]. Texas A&M University; 2006. Available from: https://core.ac.uk/download/pdf/4278102.pdf.

[99] Mangundjaya WLH. Is there cultural change in the national cultures of Indonesia? In: Kashima Y, Kashima ES, Beatson R, editors. Steering the cultural dynamics: Selected papers from the 2010 Congress of the International Association for Cross-Cultural Psychology. 2013. Available from: https://scholarworks.gvsu.edu/iaccp_papers/105/.

[100] French DC, Rianasari M, Pidada S, Nelwan P, Buhrmester D. Social support of Indonesian and U.S. children and adolescents by family members and friends. Merrill Palmer Q. 2001;47(3):377–94. Available from: https://www.jstor.org/stable/23093404.

[101] Adnyani NLPS, Suwastini NKA. Language and power in various social contexts. In: Proceedings of the 2nd International Conference on Languages and Arts across Cultures (ICLAAC 2022). Paris: Atlantis Press SARL; 2023. p. 86–95. Available from: https://www.atlantis-press.com/article/125978232.pdf.

[102] Harianja RF, Sudrajat A. The local wisdom of Batak Toba through the philosophy of Dalihan Na Tolu in a kinship environment. Budapest international research and critics in linguistics and education (BirLE). Journal. 2021;4(2):759–65.

[103] Muda I, Suharyanto A. Analysis of life’s inter-religious harmony based on the philosophy of Dalihan Na Tolu in Sipirok Sub-district, South Tapanuli Regency, North Sumatera Province. J Hum Behav Soc Environ. 2020;30(5):533–40.

[104] Hofstede G. Dimensionalizing cultures: The Hofstede Model in context. Online Readings in Psychology and Culture. 2011;2(1):8–26.

[105] Duijster D, Verrips GHW, van Loveren C. The role of family functioning in childhood dental caries. Community Dent Oral Epidemiol. 2014;42(3):193–205. 24117838 10.1111/cdoe.12079

[106] Guenther KD, Van Dyk TR, Kidwell KM, Nelson TD. The moderating role of dysfunctional parent-child relationships on the association between outward anger expression and physical health in youth from low-income families. J Pediatr Health Care. 2016;30(4):366–73. 26602110 10.1016/j.pedhc.2015.09.007

