# Supplementary material for: The meaning of marriage, the meaning of family, and the function of family for Indonesian married people
Source: BMC Psychol. 2025 Nov 25;13:1410. doi: 10.1186/s40359-025-03670-4 (PMC12751331; doi:10.1186/s40359-025-03670-4)
Supplement: Supplementary file 1 — Supplementary Material 1. [file 40359_2025_3670_MOESM1_ESM.doc]

**Introduction**

Greetings. Researcher introduce herself and doing small talk to build a good rapport*.*

I will clarify your personal data according to the information filled in the Google form (occupation, last education, age, age at marriage, number of children, age of first child, who you live with at home, ethnicity, religion and place of residence).

Before we start, I would like to ask for your permission to record this session. The purpose of the recording is to make it easier for the research team to transcribe and analyse the data. Do you mind if I record this meeting?

Thank you for agreeing to take part in this study. The purpose of this study is to gain insight into Indonesian marriage and family life. We are talking about your understanding and experiences of marriage and family life. I will ask you one question at a time. If there is something I do not understand, I will also ask you more questions about your answers.

Subjects who can be participants in this study are: (1) married, (2) living with their partner in the same house, (3) minimum aged 19 years old, and (4) able to use Google Meet video conferencing during the interview session.

Your participation in this research is voluntary. You can stop at any time if you feel uncomfortable. There are no significant risks to you from taking part in the interview. However, if you feel uncomfortable answering any questions, please let me know immediately so that I can move on to other questions or stop the interview.

Your participation in this study is anonymous; don't worry, we will mask your identity when processing and reporting the research results. The confidentiality of your data will also be maintained by carefully storing files and folders of recordings and transcripts of conversations, which will only be accessible to the research team.

The interview in this session will last 60-90 minutes. If you feel you need a break in the middle of the interview, please let me know. By taking part in this study, you will be contributing to research on marriage and family in Indonesia; and you may gain insight into your experiences with your family. You will be paid no later than 3 days after the interview.

Is there anything else you would like to ask about these explanations?

Before we start, I need to ask you about your willingness to participate in this research, that is, to take part in this interview session. Are you willing to participate in this study?

**Main session**:

At this meeting I would like you to share your experience and understanding of the following issues:

1. What is the meaning of marriage for you?
2. What is the meaning of family for you? Who do you think we can call family?
3. What are the functions of the family? What must the family do for its members?

Follow-up questions were asked to elicit the experience of the participant in greater depth.

**Closing**:

Thank you for your willingness to share your experiences. The thoughts and experiences you have shared with us have been invaluable to this research. I hope you will also benefit from this conversation. We will transfer the IDR 100,000 incentive no later than 3 days from now. That concludes this interview session. Thank you very much. I will stop the recording.
